# Food Grains of India

**Published:** 1858-01

**Authors:** 


### MISCELLANEA.

### FOOD-GRAINS OF INDIA.

Dr. Forbes Watson, of the East India Medical Service, has recently read at the Society of Arts a most interesting and important paper on the above-named subject; from which we take a few notes. There are in India certain vegetable substances which, according to caste, various classes are permitted to eat,—such as eggs, fish, and flesh of certain animals; but Dr. Watson, in this communication, confines his remarks to the relative nutritive value of the cereals and pulses, two or more of which, combined, constitute the entire bulk of the solid food eaten by the Brahminical or strict vegetable eating class.

Experience has taught the native how to mix his food-grains—cereal or pulse—so as to secure a true mixed food, fitted at once for the plastic and for the respiratory demands of the economy. To his rice, for respiration, he adds from one-fourth to a fifth of some leguminous seed, his favourite one the *Cajanus Indicus.* The pulses, therefore, are to the Brahmin what beef and other fleshes are to us.

Assuming six to one as the proportion between the carbonaceous and the nitrogenous foods as the requirements of the human system, Dr. Watson very ably shows that for the body to live well, this standard must be approached; that nitrogenous food cannot be applied to the plastic wants of man, unless there is a corresponding proportion of carbonaceous food also supplied; and that the theory which assigns to different substances a value in proportion to their nitrogenous compounds is fallacious.

In a series of elaborate tables, Dr. Watson epitomises the nutritive values of numerous Indian cereals and pulses. He suggests that the *Cajanus Indicus* and "*gram*"—the great horse food of India—two pulses, should be introduced into this country.

In the conclusion of his paper, Dr. Watson refers to two Eastern vegetable products, a knowledge of which is so important that we copy from him *verbatim.*

"The first of these is the *Soja hispida,* called *Bhoot* by the natives, and which I find to contain the enormous quantity of upwards of 46 lbs. per hundred of nitrogenous matter, nearly 12½ pounds of oil or fat, about 13 ounces of phosphorus, one ounce and a quarter of sulphur, and equivalent to nearly half an ounce of iron.

"With regard to it, I have been able to obtain but little general information. In Simmonds' work on the commercial products of the vegetable kingdom, it is referred to as a source of the well-known 'Soy sauce;' but if it could be grown and procured in quantity, as an addition to other food, it probably, for feeding purposes, would be found to exceed in value that of any vegetable substance now known.

"The other product, to which I would briefly refer, is marked as having come from Sumatra. It bears the native name of *Catjang tahoo,* and is probably a Dolichos bean, like the former.

"Of it I have, likewise, been able to obtain only meagre information. In Mr. Simmonds' work, a leguminous plant is mentioned, which furnishes, on pressure, a residuum known under the name of *Tanping,* and which, in the form of a dry paste, is brought to Shanghai, in a quantity amounting in value to two and a half millions sterling. This last fact is stated on the authority of the Rev. Mr. Medhurst, of Shanghai, and Mr. Thoms, British Consul at Ningpo. The bean referred to is stated as being imported into China,—pressed for its oil, which is used both for eating and burning, and the residuum, in form like large cheeses, is said to be distributed about China in every direction, and used as food for pigs and buffaloes, as well as for manure.

"Although I am not in a position positively to state that the bean which I produce this evening is identical with that referred to, still its composition leads me to infer that, if not the same article, it is one perhaps equally worthy of the attention of the agriculturists of this country. On analysis, I find that it contains per 100 lbs. weight, 39.39 lbs. of nitrogenous matter: 17 lbs. 12 oz. of oil or fatty matter; about 9½ ozs. of phosphorus and nearly two of sulphur."

Our readers will go with us in recording unanimous thanks to Dr. Watson for this great addition to the science of everyday life. It is a home question which he has discussed, and Epicurus is put in the shade. We find that the East India Company Directors assisted Dr. Watson in the pecuniary weight of this inquiry, and as this is greatly to their credit, it ought to be widely known. We hope that they will not stop in so good a work, but encourage their scientific officer to renewed and continued exertions. Such results, as they may thus by a stroke of foresight secure, are not for the good of individual men, but for nations; and what may be considered now as one of their least as well as last acts, may ultimately prove the greatest and best.

